# Deficit in Complex Sequence Processing after a Virtual Lesion of Left BA45

**DOI:** 10.1371/journal.pone.0063722

**Published:** 2013-06-10

**Authors:** Emeline Clerget, Michael Andres, Etienne Olivier

**Affiliations:** 1 Institute of Neuroscience, Laboratoire de Neurophysiologie, Université catholique de Louvain, Bruxelles, Belgique; 2 Faculty of Psychology, Department of Experimental Psychology, Ghent University, Ghent, Belgium; University of Montreal, Canada

## Abstract

Although the contribution of Broca's area to motor cognition is generally accepted, its exact role remains controversial. A previous functional imaging study has suggested that Broca's area implements hierarchically organised motor behaviours and, in particular, that its anterior (Brodmann area 45, BA45) and posterior (BA44) parts process, respectively, higher and lower-level hierarchical elements. This function of Broca's area could generalize to other cognitive functions, including language. However, because of the correlative nature of functional imaging data, the causal relationship between Broca's region activation and its behavioural significance cannot be ascertained. To circumvent this limitation, we used on-line repetitive transcranial magnetic stimulation to disrupt neuronal processing in left BA45, left BA44 or left dorsal premotor cortex, three areas that have been shown to exhibit a phasic activation when participants performed hierarchically organised motor behaviours. The experiment was conducted in healthy volunteers performing the same two key-press sequences as those used in a previous imaging study, and which differed in terms of hierarchical organisation. The performance of the lower-order hierarchical task (Experiment #1) was unaffected by magnetic stimulation. In contrast, in the higher-order hierarchical task (Experiment #2, “superordinate” task), we found that a virtual lesion of the anterior part of Broca's area (left BA45) delayed the processing of the cue initiating the sequence in an effector-independent way. Interestingly, in this task, the initiation cue only informed the subjects about the rules to be applied to produce the appropriate response but did not allow them to anticipate the entire motor sequence. A second important finding was a RT decrease following left PMd virtual lesions in the superordinate task, a result compatible with the view that PMd plays a critical role in impulse control. The present study therefore demonstrates the role of left BA45 in planning the higher-order hierarchical levels of motor sequences.

## Introduction

While a large variety of cognitive tasks, including motor tasks, leads to an activation of Broca's region, the exact meaning of this possible overlap remains debated (e.g., [1]). One possible explanation is that Broca's region contains several small functional subunits, explaining this apparent overlap between activations elicited by motor and cognitive tasks [2], [3]. Alternatively, it has been suggested that Broca's area is active in an extensive number of tasks because this area is responsible for implementing a process common to all of them. Accordingly, it has been suggested that Broca's region might act as a “supramodal syntactic processor”, able to process any type of hierarchically organised sequences [4], [5], a hypothesis rooted in the finding that this region is not only involved in processing language syntax (e.g., [6]) but also syntax-like aspects of non-linguistic tasks (see [7], [8]).

The role of “syntactic processor” has been assigned either to the posterior (BA44) [9]–[11] or anterior part (BA45) [12]–[15] of Broca's area. A possible explanation for this divergence about the role of BA44 and BA45 in syntax processing is that their contribution might depend on the hierarchical level of the element to be processed (e.g., [16]). A similar assumption has been made for motor behaviours by Koechlin and Jubault [17] who proposed a model in which the anterior (BA45) and posterior (BA44) parts of Broca's area constitute, with the dorsal part of the premotor cortex, a rostro-caudally organized network for processing hierarchically structured sequences, in which the most rostral area, BA45, processes the hierarchically higher elements of the motor plan. One key assumption of that model is that motor behaviour shares some similarities with language, namely that a complex action can be viewed as a chain of subordinate movements, which need to be combined according to certain rules in order to reach a given goal [18]–[20]. The model of Koechlin and Jubault [17] was build on the basis of functional imaging data gathered in two motor tasks characterized by different hierarchical levels of organization. In a first task, called “simple task” by Koechlin and Jubault [17], subjects had to execute “simple action chunks” which consisted in performing a pre-learned sequence of button-presses, regarded as single motor acts. In contrast, in a so-called “superordinate task”, subjects had to select amongst three possible “simple action chunks” corresponding to three distinct pre-learned rules associating a given cue (a letter) to a given button-press. This is reminiscent of the three hierarchical levels proposed by Dehaene and Changeux [18] for sequence planning, namely (i) elementary gestures, (ii) an operation combining several elementary gestures and (iii) a planning system. Based on distinct phasic activations of BA6, BA44 and BA45 during the performance of these two motor tasks, Koechlin and Jubault [17] concluded that Broca's area process hierarchically structured behaviour, a view applicable to other cognitive functions, including language.

However, despite the fact that this conclusion is in accordance with numerous studies related to the functional organisation of the frontal cortex [20]–[27], it does not allow us, because of the correlative nature of functional imaging data, to determine unambiguously the causal role of BA6, BA44 and BA45. To circumvent this inherent limitation of functional imaging results, here we used on-line repetitive transcranial magnetic stimulation (rTMS) to disrupt transiently the neural processing in the three aforementioned areas in the left hemisphere. Although the study of Koechlin and Jubault [17] also reported some activation in the right hemisphere, here we decided to focus on left frontal areas because of the well-known left hemispheric dominance for language and because left hemisphere frontal areas have been more often found implicated in action-related tasks (e.g., [28]).

## Materials and Methods

### Experiment #1: simple task

#### Participants

Seven subjects (27±4 years) participated in Experiment #1. They were all right-handed, as assessed by the Edinburgh handedness inventory [29]; they all had normal, or corrected to normal, vision and no neurological disease history; none of them was under the influence of medication, alcohol or drug. Each subject was seen by a neurologist to rule out potential risk of adverse reactions to TMS, based on the Transcranial magnetic stimulation Adult Safety Screen (TASS; [30]). The study was carried out according to the Declaration of Helsinki. All subjects gave their written informed consent and were compensated for their participation. The Ethics Committee of the Université catholique de Louvain has approved all experimental procedures.

#### Task

**Figure 1:**
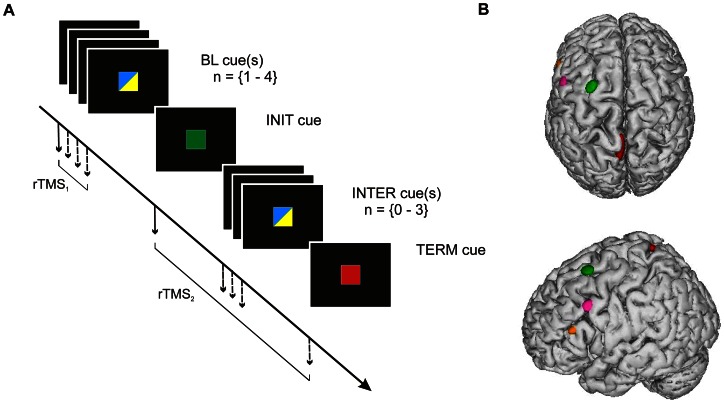
Trial time course and TMS target sites. A. Time course of cue presentation in both tasks. A trial always started by the presentation of 1–4 baseline (BL) cue(s), which were either blue or yellow; it was followed by one green initiation (INIT) cue, 0–3 intermediate (INTER) blue or yellow cue(s) and a red termination (TERM) cue (see Methods and Fig. 2 for further detail). Each of the cues was displayed for 500 ms and was either a square in Experiment #1 (as shown on this Figure) or a letter (A, B or C) in Experiment #2. The delay between each cues was varied randomly, by step of 500 ms, from 2500 to 4000 ms. Two TMS trains (3 pulses at 10 Hz) were delivered during a given trial: one TMS train (rTMS_1_) was delivered at the onset of one of the BL cues, a second train (rTMS_2_) was delivered at the onset of either the INIT, INTER or TERM cue, as indicated by the arrows. B. Location of the TMS sites. The TMS sites are shown as ellipses on a lateral and superior views of a normalized brain. Each ellipse is centred on the mean MNI coordinates of the corresponding stimulation site (PMd in green, PB in pink, AB in orange and the control site in red). The surface of each ellipse indicates the 95% confidence interval of the normalized coordinates average for all subjects (n = 16).

**Figure 2:**
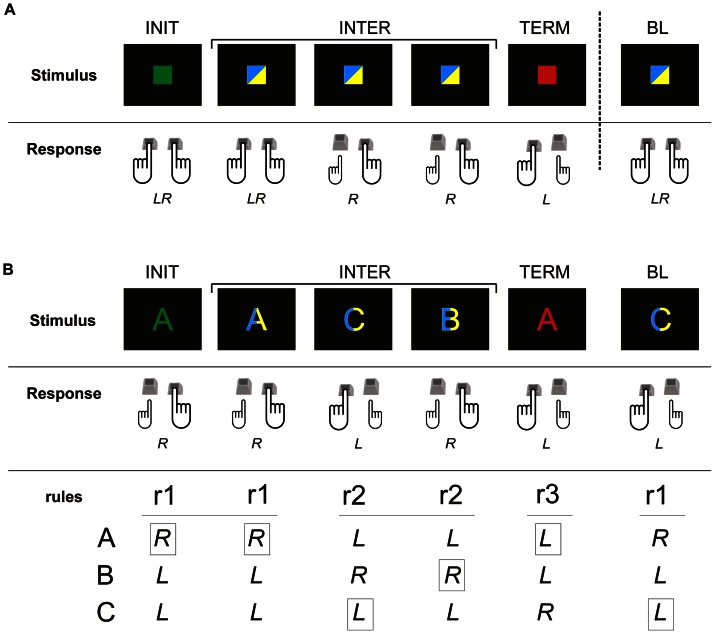
Stimulus-response mapping in both tasks. In both tasks, the green and red cues were instructive cues, indicating respectively the beginning (INIT cue) and the end (TERM cue) of a sequence. INTER and BL cues were either blue or yellow, and are shown as bicolour on this figure for the sake of simplicity. The motor response to those different cues was a flexion of either the right (R), left (L) or both (LR) index finger(s) performed by pressing, respectively, the left, right or both Ctrl key(s) on a computer keyboard. A. Stimulus-response mapping for the simple task (Experiment #1): the cues were squares and each of the cues was associated with a specific motor response (INIT: LR, first INTER: LR, second and third INTER: R, TERM: L and BL: LR). B. Stimulus-response mapping for the superordinate task (Experiment #2): in this task, the cues were letters (A, B or C). Each of the cues was no longer associated with a given motor response but with a given rule (r1, r2 or r3) (INIT: r1, first INTER: r1, second and third INTER: r2, TERM: r3 and BL: r1) and, for each rule, the letter determined the motor response to be executed. It explains both the association between the letters and the correct motor response for each rule, and the response to be chosen (indicated by the black rectangles) in the example depicted.

The task used in Experiment #1 (see Figure 1A and Figure 2A) corresponded to the “simple task” designed by Koechlin and Jubault [17]. It consisted of executing a pre-learned sequence of key-press movements performed either with the left (L), the right (R) or both index fingers (LR). In this experiment, the sequence of key-presses was always the same, namely LR, LR, R, R, L. A green square displayed at the centre of a computer screen triggered the beginning of a new sequence, indicating that subjects had to generate the first movement (LR); this green square was named the initiation (INIT) cue and was displayed for 500 ms, like the other cues. The next movements of the sequence (LR, R, R) were triggered by a so-called intermediate (INTER) cue, being, randomly, either a blue or a yellow square, and displayed at an interval varying between 2500 and 4000 ms, incremented by steps of 500 ms. These intervals were shorter than those used in the original experiment [17] in order to reduce the experiment duration, but were long enough to ensure a sufficient delay between two TMS trains. The end of the sequence was indicated by the presentation of a red square, so-called the termination (TERM) cue, used to trigger the last movement (L); the TERM cue could either be displayed after the completion of a full sequence (endogenous termination) or, unpredictably, during the course of a sequence (exogenous termination). The proportion of endogenous and exogenous terminations was identical and the three possible cases of exogenous termination (after the INIT cue or after the first or the second INTER cue) were equally distributed. In order to gather a baseline reaction time (RT) for movements not performed inside a sequence, the subjects performed LR movement(s) triggered by baseline (BL) cues, which were either blue or yellow squares, displayed in-between two sequences; 1–4 BL cues were presented before each sequence, at a variable delay (2500 to 4000 ms, step of 500 ms).

Overall, one trial consisted of either one complete or one aborted sequence followed by 1 to 4 baseline movements, its duration varied approximately between 9 and 27 s.

#### Experimental procedure

The experiment was divided into three sessions, spread over one week. The first session was a training session in which the subjects repeated the task until they reached 95% of correct trials. This training session usually consisted in 4 blocks of 15 trials. The two following sessions were TMS sessions (see below), each of them being composed of 4 blocks of 15 trials. During each block, TMS was applied over one of the four stimulation sites investigated in the present study (see below). The block order was randomized across subjects but was kept constant for each subject across sessions.

#### Transcranial magnetic stimulation

The TMS was delivered through a 70 mm figure-of-eight coil connected to a Rapid Magstim model 200 stimulator (Magstim Company, Whitland, UK). During the first training session, the resting motor threshold (rMT) for the hand representation of left primary motor cortex was evaluated for each subject. The rMT was defined as the minimum TMS intensity necessary to induce 50 µV peak-to-peak motor evoked potentials (MEPs) in the first dorsal interosseus muscle in about 5 out of 10 trials. The TMS intensity was set at 110% of individual rMT. Pulses were delivered at 10 Hz during 200 ms and were synchronized on the cue onset (3 pulses at 0, 100, 200 ms with respect to the cue onset). To comply with the safety guideline [31], only 2 rTMS trains were delivered in each trial, one synchronized with the onset of either an INIT, INTER or TERM cue, and the second one with the display of a BL cue; each trial was designed in such a way that rTMS was never delivered on two successive cues and were separated by at least 5 s. The cues not followed by an rTMS train (“no TMS trials”) served as a control to investigate RT changes in response to different cues, and therefore two types of control trials were available in the present study: 1) “no-TMS trials” and 2) TMS trials gathered following the control stimulation site (see below).

#### Location of stimulation sites

The coil position was precisely guided by means of a neuronavigation technique [32]–[35] used to locate stimulation sites onto individual anatomical magnetic resonance images previously gathered for each subject. The targeted sites were the three areas in the left hemisphere in which Koechlin and Jubault [17] reported a phasic activation during the simple and superordinate tasks, namely the posterior (PB) and anterior (AB) parts of Broca's region and the dorsal premotor cortex (PMd). In each subject, the PMd, PB and AB sites were determined by using a “probabilistic” approach (for further details, see [36]). To target PB (target coordinate: −44, 2, 39 mm) and AB (target coordinate: −44, 24, 19 mm) we used the coordinates of the activation peaks reported by [17]. To estimate the localization of PMd (target coordinate: −25, 4, 72 mm, Montreal Neurological Institute (MNI) system of coordinates), we averaged the coordinates reported in three previous TMS experiments that have investigated the role of left PMd in action selection tasks [37]–[39]. These sites were transformed into the individual subject brain coordinates using a reverse normalization procedure and were displayed on the MR image of each subject. Then, the accurate positioning of the coil onto the scalp was ensured by using a home-made neuronavigation program [40].

The leg representation in the left primary motor cortex (M1_leg_) was used as a control site. The location of M1_leg_ was determined in each subject by searching the point that produced an observable movement of the right leg.

Finally we performed an off-line normalization of individual coordinates of the TMS sites with respect to the MNI brain atlas to establish the coordinates of the actual TMS sites, with respect to the targeted coordinates. The normalization procedure was performed by normalizing each individual (native) head image to the standard MNI brain template by mean of an iterative algorithm that searches for the optimal projection of any individual brain onto the MNI brain. Details about these neuronavigation and normalization procedures are available elsewhere [32], [37], [40]. The mean coordinates (mean±SD for each coordinates x, y and z, n = 16) of the four stimulated areas were as follows: M1_leg_ (−4±5, −24±22, 74±6 mm); PMd (−28±4, 10±2, 65±4 mm); PB (−55±3, 10±8, 43±3 mm) and AB (−56±3, 27±5, 23±4 mm) (Figure 1B).

#### Data acquisition and statistical analysis

The experiment was implemented with Matlab (The Mathworks, Inc.) running on a personal computer. Reaction time was defined as the delay between the cue onset and the corresponding key-press; RT was computed separately for each cue. Incorrect trials (2.45±1.28%) and trials in which RT was larger than the mean individual value ±2 standard deviations for each subject were discarded (4.71±1.25%) from the subsequent analyses.

First, for each subject and each rTMS condition, we measured the relative RT changes, expressed in percentage, between TMS and no-TMS trials: RT change  =  (RT_TMS_ – RT_noTMS_)/(RT_noTMS_) * 100. Then, in order to exclude unspecific TMS effects (noise, tactile sensations…), we expressed these values with respect to values gathered for M1_leg_. To do so, we computed the difference between the RT changes obtained for each experimental site (AB, PB, PMd) with the RT changes gathered for M1_leg_. This computation provided us with the “specific TMS effect”. Normality tests indicated that the specific TMS effect values followed a Gaussian distribution (Kolmogorov-Smirnov d = 0,07, p =  n.s.; Chi-Square test  = 5,66, df  = 3 (adjusted), p = 0,13).

Data were analysed by using repeated measures ANOVA (ANOVA_RM_). To determine the subject performance in control (no-TMS) trials, we performed an ANOVA_RM_ on RT with the CUE (BL, INIT, INTER, TERM) as a within factor. The RT change was analysed by mean of a one-way ANOVA_RM_ with TMS (no TMS, TMS) as a within factor. Finally, to demonstrate a specific TMS effect on RT for the different cues and stimulation sites, we performed a two-way ANOVA_RM_ with the SITE (PMd, PB and AB) and CUE (BL, INIT, INTER, TERM) as within factors. It is noteworthy that the distinction between endogenous and exogenous termination trials was not made in this analysis since no RT difference was found between these two conditions.

When appropriate, post-hoc comparisons were performed using a Tukey test, except for the analysis of the specific effect of TMS for which we used a Dunnett test, the most appropriate test to reveal significant differences between the BL cue and every other cues as this test is favoured when the mean has to be compared with a standard reference.

### Experiment #2. Superordinate task

The aim of Experiment #2 was to address the same issue as in Experiment #1 but in a more complex task, named “superordinate task” by Koechlin and co-workers [17]. Because both Experiments #1 and #2 were identical in many aspects (TMS application, stimulation sites, data acquisition, analyses, …), only the points that differed from Experiment #1 will be described in the following sections.

#### Participants

Sixteen subjects took part in Experiment #2, five of them having participated in Experiment #1. Two subjects were excluded from the subsequent analyses because either their mean RT (subject #2) or error rate (subject #13) was larger than the mean group value +2 SD. The mean age of the 14 remaining subjects was 27±4 years.

#### Task

In the “superordinate task” (Figure 2B), the square cues were replaced by one of three possible letters (A, B, or C) displayed pseudo-randomly, while both the timing and colour code remained the same as in Experiment #1: green and red letters represented the INIT and TERM cues, respectively, while the blue and yellow letters indicated either INTER or BL cues. As in Experiment #1, the subjects had to perform a key-press task but, in this experiment, the responses differed across trials depending on three rules (r1, r2, r3), which associated a given letter with a given key-press (see Fig. 2B). Therefore, the INIT cue triggered the application of a sequence of pre-learned rules (r1, r1, r2, r2, r3) instead of a sequence of pre-learned key-presses, as in Experiment #1 (Figure 2B). Because the actual movement triggered by a given letter was different for each rule, this task ensured that each trial was different in terms of key-press sequence to be performed. Indeed, the r1 rule associated an “A” with a right index key press whereas “B” and “C” were associated with a left index movement. The r2 rule associated the “B” with a right key press, “A” and “C” with a left key press and in r3, “C” was associated with a right finger response and “A” and “B” with a left one (Figure 2B). In a given block of 15 trials, letters were pseudo-randomly chosen so that the proportion of left and right responses was identical. In response to BL cues, participants were instructed to apply always the r1 rule.

As in Experiment #1, incorrect trials (3±2%) or trials with RT longer than the individual mean ± 2 SD were discarded (4.79±0.74%) from analyses.

#### Data acquisition and statistical analysis

The specific TMS effect was computed as in Experiment #1 (see above) and an additional ANOVA_RM_ was performed on significant specific TMS effect in order to investigate whether these effects differed as a function of the hand (left vs right) used to respond to a given cue.

## Results

### Experiment #1: Simple task

**Figure 3:**
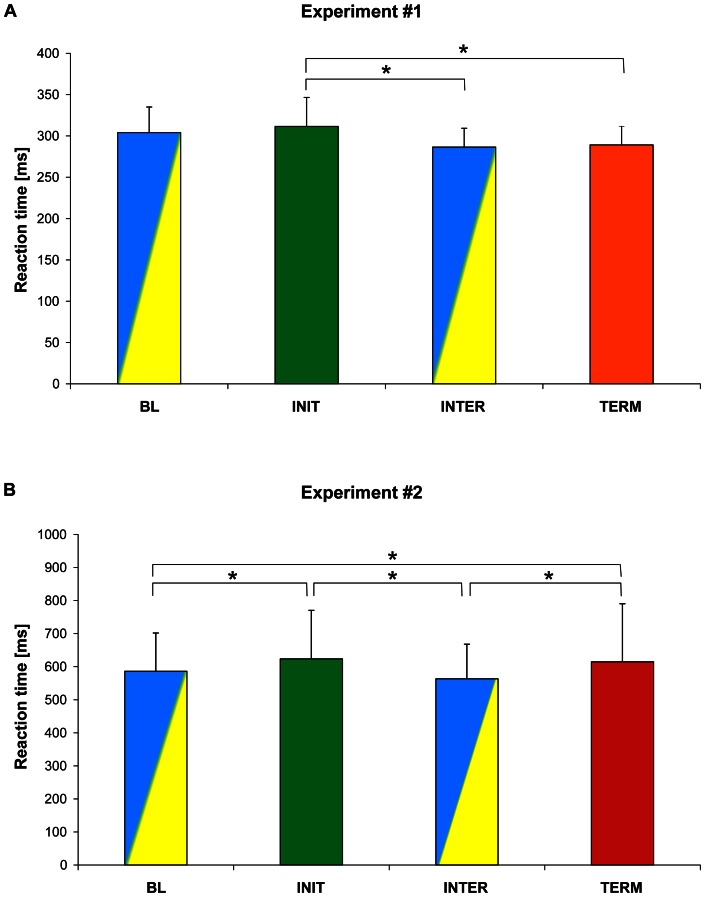
Reaction times for each type of cues in the control condition. Mean reaction times (± SEM) for all subjects, in the absence of TMS, for each type of cues (BL: baseline, INIT: initiation, INTER: intermediate, TERM: termination). The code of colour is the same as that used for the cue in the experiments. A. In the simple task (Experiment #1), RT were significantly larger for the INIT cues than for the INTER and TERM cues. B. In the superordinate task (Experiment #2), RT were significantly longer for the INIT and TERM cues than for the BL and INTER cues.

An ANOVA_RM_ performed on no-TMS trials with CUE as a within factor revealed a main effect of the CUE on RT (F_3,18_ = 6.78, p<0.003) (Figure 3A). Post-hoc comparisons showed that RT was significantly longer for the INIT cue (311±35 ms, mean ± SD, n = 7) than for INTER cues (286±23 ms, p = 0.002) and TERM cues (289±22 ms, p = 0.004). This finding corroborates the well-known observation that the initiation of a new sequence is more demanding than processing the subsequent cues (add ref on chunking), and confirms the effectiveness of the task manipulation. However, in contrast to Koechlin and Jubault's results, we failed to find an increased RT for TERM cues.

A one-way ANOVA_RM_ performed on all trials revealed a main effect of TMS on RT (F_1,6_ = 141.34, p<0.001) showing that in the TMS condition, mean RT (236±33 ms) was about 20% (60 ms) shorter than in the no-TMS condition (296±27 ms). This RT decrease reflects an arousal effect induced by the artefacts of TMS [41].

In this experiment, the two-way ANOVA_RM_ (SITE × CUE) did not show a main effect of the SITE or CUE, or an interaction between these factors (all F<1.2, all p>0.3).

### Experiment #2: Superordinate task

As in Experiment #1, we found a main effect of the CUE on RT in no-TMS trials (ANOVA_RM_, F_3,39_ = 16.03, p<0.001) (Figure 3B). Post-hoc comparisons confirmed that RT to the INIT cues (624±147 ms, mean ± SD, n = 14) was significantly longer than RT to the BL (586±116 ms, p = 0.002) and INTER cues (563±105 ms, p<0.001). In line with Koechlin and Jubault's results, we also found that RT for the TERM cues (615±131 ms) was significantly longer than responses to BL cues (586±116 ms, p = 0.027) and to INTER cues (563±105 ms, p<0.001).

The main effect of TMS on RT (one-way ANOVA_RM_; F_1,13_ = 36.33, p<0.001) indicated that, in the TMS condition, the RT (559±119 ms) decreased by about 5% (30 ms) when compared with the no-TMS condition (587±117 ms) (p<0.001), seemingly due to the unspecific TMS effect.

**Figure 4:**
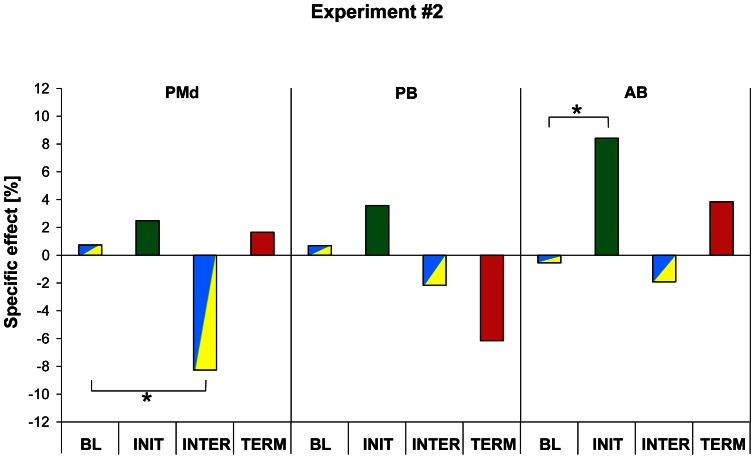
Specific effect of TMS in the superordinate task (Experiment #2). For each type of cues, the relative TMS effect obtained for the control site was subtracted from the TMS effect obtained for the three targeted sites (PMd: dorsal premotor cortex, PB: posterior part of Broca's area, AB: anterior part of Broca's area), allowing us to calculate a “specific TMS effect” (see Methods for detail). A positive value indicates that TMS over one of these targets led to an increase in RT when compared to the TMS effect observed for the control site, a negative value indicates a decrease in RT. Significant “specific TMS effects” were found following PMd and AB virtual lesions, leading, respectively, to a decrease in RT for the INTER cues and an increase in RT for the INIT cues.

The specific TMS effect for each stimulation site (PMd, PB and AB) is illustrated in Figure 4: positive values indicate that TMS led to an increase in RT when compared to M1_leg_ stimulation site whereas negative values indicate shorter RT. The ANOVA_RM_ showed a significant SITE × CUE interaction for RT (F_6,78_ = 2.53, p = 0.027) and post-hoc comparisons indicated that virtual lesions of PMd led to a shorter RT only for movement performed in response to INTER cues (p = 0.036). In contrast, we found that a virtual lesion of AB yielded a slowing down of responses to the INIT cues (p = 0.038). Virtual lesions of PB had no effect on cue processing in the superordinate task.

In order to investigate further these effects of TMS, we performed an additional ANOVA_RM_ taking into account the HAND factor (see Methods). This analysis showed a main effect of HAND (F_1,9_ = 5.42, p = 0.045, indicating that, overall, the specific TMS effect was larger for the responses performed with the right index than with the left index (p = 0.045). This ANOVA_RM_ also showed a SITE × CUE × HAND interaction (F_6,54_ = 3.06, p = 0.012) and the post-hoc comparisons indicated that the decrease in RT found after a virtual lesion of PMd for INTER cues was caused by a decrease in RT of responses performed with the right, contralateral, index finger, the left index responses being indistinguishable from the baseline in terms of RT. the TMS effect found for the INIT cues following AB virtual lesions was not related to a particular hand (p = 0.72).

## Discussion

The main goal of the present study was to determine the causal role of Broca's area in processing hierarchically structured motor sequences, and to attempt to discriminate the specific contribution of its anterior (BA45) and posterior (BA44) parts. To address this issue, we applied TMS over AB (BA45) and PB (BA44), in which a singular phasic activation was reported in subjects executing distinct motor sequences characterized by various levels of hierarchical organization [17]. This finding led Koechlin and Jubault [17] to build a model postulating that three frontal areas, including AB and PB, arranged along a rostro-caudal gradient, process the different hierarchical levels of structured behaviours. Hence, this model predicts that the effect of a virtual lesion of AB or PB, as induced by rTMS, should vary for the different cues and different tasks. In the simple task, this model predicts that PB virtual lesions should disturb the processing of the INIT and TERM cues and, in the superordinate task, that of INTER cues, because these different cues represent, in both tasks, transitions between “simple action chunks”. Furthermore, it predicts that AB virtual lesions should have no effect on the simple task performance but should delay the initiation and termination of superordinate chunks.

One key finding of the present study is that, in Experiment #2, virtual lesions of left AB selectively delayed the processing of the INIT cue which leads the superordinate chunks, but had no effect on the processing of other cues; rTMS applied over left AB had no consequence on task performance in Experiment #1, as predicted by Koechlin and Jubault's model [17]. Because this effect was specific for the INIT cue of the superordinate chunks, we can rule out that rTMS might interfere with other higher cognitive processes, such as working memory. This also allows us to rule out that the effects reported in the superordinate task, and not in the simple task, was due to a difference between task difficulties, otherwise the same effect should have been found for all cues of the superordinate task, in particular for the TERM cues leading to RT equivalent to that for the INIT cues (see below). This finding therefore supports the specific role of left AB in processing hierarchically higher levels of action plans. Importantly, in the superordinate task, the INIT cue was not connected to a unique sequence because the design of this task yielded different motor responses in each trial; instead, the INIT cue prompted the retrieval and application of a pre-learned sequence of rules (r1, r1, r2, r2, r3). Along the same lines, we found that, in the superordinate task, the effect of left AB virtual lesions on the INIT cue processing was not hand-specific. This constitutes another argument favouring the view that left AB is causally involved in an abstract operation analogous to the “plan level” suggested by Dehaene and Changeux [18], and that its contribution is therefore effector-independent.

The model of Koechlin and Jubault [17] made other predictions that we failed to confirm in the present study. First, in the “superordinate task”, AB virtual lesions should have also disrupted the processing of the TERM cues, a prediction that we did not confirm here. Indeed, Koechlin and Jubault [17] reported a phasic activation in left AB at both boundaries or superordinate chunks, i.e. for the INIT and TERM cues. The conclusion of these authors was that AB, in addition to its contribution to complex sequence initiation, is also involved in ending superordinate chunks via top-down interactions that prevent the sequential selection of superordinate chunk components, i.e. the three potential “simple action chunks” called by each letter. This TMS effect was lacking despite the fact that the processing of TERM cues led to a larger RT, confirming the effectiveness of the task manipulation; this RT increase indicates that the TERM cues were treated distinctively and that they mobilized additional resources. One possible explanation for the lack of AB virtual lesion on TERM cue processing is that the ending of superordinate chunks relies on a more distributed network.

Another essential prediction made by Koechlin and Jubault [17], and that we failed to corroborate, is that left PB processes the transition between “simple action chunks”, a process comparable to the intermediate “operation level” defined by Dehaene and Changeux [18]. This implies that, in the simple task, PB virtual lesions should have altered the processing of INIT and TERM cues and that of INTER cues in the superordinate task, a prediction that we failed to confirm. The absence of effect of PB virtual lesions remains puzzling, although the equivalence of these different cues in the two tasks developed by Koechlin and Jubault [17] could be questioned. Indeed, we have already demonstrated the causal role of left BA44 in motor and perceptive chunking [34], [42], in agreement with some functional imaging studies [43] and, recently, Wymbs et al. [44] have proposed that Broca's region is part of a left fronto-parietal network responsible for segmenting sequences into multiple chunks. However, while interpreting the present results, it is important to keep in mind that in the original experiment of Koechlin and Jubault [17], their definition of “chunk” was at odds with that usually used in the literature and that processing the INIT and TERM cues in the “simple task” and processing the INTER cues in the “superordinate task” are unlikely to rely on the same cognitive processes. Indeed, chunking is usually regarded as a strategy used for enhancing performance when learning complex sequences [45] and thought to comprise two processes 1) a segmentation phase which consists in breaking apart a sequence into smaller groups of elements or “chunks” and 2) a concatenation phase, thought to occur later on when learning a sequence, and which consists in chaining these chunks together so that the sequence can be performed as a unified action [44]. Nonetheless, based on functional magnetic resonance imaging results of Koechlin and Jubault [17], left BA44 was similarly activated in the simple and superordinate tasks, while processing, respectively, the INIT and TERM cues, and the INTER cues, and, therefore, rTMS applied over left PB should have influenced the RT.

One possible explanation for the discrepancy between the prediction of the model of Koechlin and Jubault [17] and the present study is the exact location of the PB stimulation site. As shown in Figure 2B, while the location of the AB site fits well with BA45, the PB site was located slightly dorsally with respect to the inferior frontal sulcus, and therefore close to the upper limit of the left BA44. It is worth noting that in our previous TMS studies investigating the contribution of left BA44 to sequence or action processing, the stimulation site was located slightly more ventrally [33]–[35]. Another possible explanation is that Koechlin and Jubault [17] reported, in both tasks, a bilateral brain activity in PMd, PB and AB, a finding consistent with other studies on the role of Broca's area in non-linguistic syntax (for instance, [46]). Therefore, it is sensible to assume that, if processing “simple action chunks” involved both BA44, TMS applied only to left BA44 may have failed to induce a noticeable behavioural deficit because the right BA44 may have compensated for the TMS effects. The question as to whether different results would have emerged following a simultaneous and bilateral stimulation of BA44 is an open question for future investigations; a similar issue has already been solved in the literature by applying TMS bilaterally to find out the contribution of AIP to grasping movements [32].

The second important result of the present study was a RT decrease found following left PMd virtual lesions for response to the INTER cues in the superordinate task. In the present study, the location of the PMd site we targeted corresponds to the dorsal part of the premotor cortex, a region known to play a role in movement selection [38], [39], [47]. We found that this decrease in RT was observable only for responses performed with the right, contralateral, hand. This effect contrasts with the aforementioned effect of AB TMS, which was effector-independent. This finding also contrasts with many previous studies which have concluded to a dominance of left PMd in movement selection, as shown by the finding that TMS application over left PMd affected performance of both hands whereas right PMd stimulation only impaired contralateral responses [38], [39], [47]. The other discrepant aspect of this finding is that rTMS applied over PMd led to a shortening of RT, in contrast to results of most TMS studies, which usually reported a decrease in performance following TMS application. It is noteworthy that a somewhat similar enhancement of performance in an action selection task induced by left PMd TMS has been previously reported, also for both hands [48]. The decrease in RT following virtual lesions of PMd is compatible with the view that this area is critically involved in the so-called “impulse control” [49]. Indeed, it is sensible to assume that, if the inhibitory signal responsible for avoiding premature movement onset originates from PMd, rTMS applied over this region disrupted the impulse control [50] and led to a RT reduction. This effect was not found in the simple task probably because the action selection process was easier, a condition likely to necessitate less inhibitory control.

In the Introduction, we pointed out that syntax processing in non-linguistic tasks was related to BA44 in some studies [9]–[11] and to BA45 in others [12]–[15]. The hypothesis originally proposed by Koechlin and Jubault [17] that there exists a gradient within Broca's area for controlling different hierarchical levels still represents an interesting way to reconcile these apparently discrepant findings although their tasks did not really manipulate the syntactic structures of the motor sequences. Interestingly, Friederici et al. [51] pointed out the existence of a similar posterior-to-anterior gradient in the prefrontal cortex for processing hierarchically structured mathematical formulae whereas the processing of complex syntactic hierarchies in language is confined in the posterior part of Broca's area. The bottom line of this model is that the posterior part of Broca's area will control highly automatic, less demanding, syntactic processing while the anterior part of Broca's area will be involved in less automatic, highly demanding, syntactic processing [51]. The superordinate task used in the present experiment clearly falls into the second category of less automatic hierarchical processing, providing another framework for explaining the implication of the anterior part of Broca's area in this task.
